# The prognostic marker FLVCR2 associated with tumor progression and immune infiltration for acute myeloid leukemia

**DOI:** 10.3389/fcell.2022.978786

**Published:** 2022-10-12

**Authors:** Xiuhua Su, Guangxin Ma, Xiaoran Bai, Juan Zhang, Mingying Li, Fan Zhang, Tao Sun, Daoxin Ma, Fei Lu, Chunyan Ji

**Affiliations:** ^1^ Department of Hematology, Qilu Hospital, Cheeloo College of Medicine, Shandong University, Jinan, China; ^2^ School of Medicine, Cheeloo College of Medicine, Shandong University, Jinan, China; ^3^ Hematology and Oncology Unit, Department of Geriatrics, Qilu Hospital, Cheeloo College of Medicine, Shandong University, Jinan, China; ^4^ Department of Critical Care Medicine, Qilu Hospital of Shandong University, Jinan, China; ^5^ Shandong Key Laboratory of Immunohematology, Qilu Hospital, Cheeloo College of Medicine, Shandong University, Jinan, China

**Keywords:** acute myeloid leukemia, FLVCR2, tumor microenvironment, prognosis, immune cell infiltration

## Abstract

Acute myeloid leukemia (AML) is one of the most common hematopoietic malignancies in adults. The tumor microenvironment (TME) has a critical effect on AML occurrence, recurrence, and progression. The gene feline leukemia virus subgroup C cellular receptor family member 2 (FLVCR2) belongs to the major facilitator superfamily of transporter protein members, which is primarily involved in transporting small molecules. The potential role of FLVCR2 in the TME in AML has not been investigated. To clarify the expression and role of FLVCR2 in AML, we analyzed the Gene Expression Omnibus and The Cancer Genome Atlas databases and found that FLVCR2 mRNA expression significantly increased among patients with AML. Furthermore, based on an analysis of the Gene Expression Profiling Interactive Analysis database, FLVCR2 upregulation predicted dismal overall survival of patients with AML. Our validation analysis revealed the significant upregulation of FLVCR2 within the bone marrow of AML relative to healthy controls by western blotting and qPCR assays. Gene set enrichment analysis was conducted to explore FLVCR2’s related mechanism in AML. We found that high FLVCR2 expression was related to infiltration degrees of immune cells and immune scores among AML cases, indicating that FLVCR2 possibly had a crucial effect on AML progression through the immune response. Specifically, FLVCR2 upregulation was negatively related to the immune infiltration degrees of activated natural killer cells, activated memory CD4^+^ T cells, activated dendritic cells, and CD8^+^ T cells using CIBERSORT analysis. According to the *in vitro* research, FLVCR2 silencing suppressed AML cell growth and promoted their apoptosis. This study provides insights into FLVCR2’s effect on tumor immunity, indicating that it might serve as an independent prognostic biomarker and was related to immune infiltration within AML.

## Introduction

Acute myeloid leukemia (AML) is a frequently observed and highly heterogeneous adult hematologic malignancy, and its features include clonal expansion, impaired differentiation, and apoptosis evasion in normal hematopoietic stem cells (Kumar, 2011; Chen et al., 2019). Therapeutic strategies for AML include traditional chemotherapy, hematopoietic stem cell transplantation, and immunotherapy. Despite the use of new therapeutic targets and drugs, the mortality rate of AML remains high. Long-term overall survival (OS) is less than 40% in patients aged under 60 years and less than 15% in patients aged over 60 years (Dohner et al., 2015). Therefore, defining the mechanisms underlying AML progression and improving the prognosis is necessary.

The causes of AML are typically related to genetic mutations and immune system deficiency. The tumor microenvironment (TME) affects AML pathogenesis and development (Carter et al., 2019; van Galen et al., 2019). TME components, such as changed extracellular matrix, suppressive immunocytes, and soluble factors, have been shown to inhibit tumor immunotherapy and promote AML progression (Ladikou et al., 2020). The immunosuppressive microenvironment mainly contains reduced natural killer (NK) cells and cytotoxic T lymphocytes and the accumulation of myeloid-derived suppressor cells, macrophages, and regulatory T cells (Tregs) (van Galen et al., 2019). Immunocyte-based therapy has gained attention as a possible approach to antitumor therapy. However, immunotherapy has shown less efficacy for AML than for other solid cancers (Austin et al., 2016; Khaldoyanidi et al., 2021). Furthermore, for AML, there is no accurate prognostic model based on the immune microenvironment. Consequently, identifying possible markers for diagnosing and treating AML to improve patient prognosis is urgently necessary.

Feline leukemia virus subgroup C cellular receptor family member 2 (FLVCR2) belongs to the major facilitator superfamily (MFS) of transporter protein members that transport small molecules (Pao et al., 1991). MFS is vital to the movement of various substrates across membranes and for lipid synthesis, which are essential for immune homeostasis (Quistgaard et al., 2016; Zhou et al., 2019; Fu et al., 2021). FLVCR2 is widely expressed in animal tissues, such as the brain, placenta, lung, liver, and kidneys (Duffffy et al., 2010). FLVCR2 transporter controls calcium entering target cells and participates in regulating calcium metabolism and development (Brasier et al., 2004). Moreover, FLVCR2 has been shown to be important for heme transport (Duffffy et al., 2010). In humans, FLVCR2 mutations are related to Fowler syndrome, the cerebral proliferating vascular disease (Meyer et al., 2010). Additionally, FLVCR2 has been demonstrated as a potential biomarker for predicting prostate cancer progression, especially at stage II (Alkhateeb et al., 2019). Evidence on the role of FLVCR2 in AML is limited, and the biological functions of FLVCR2 in AML remain unclear; thus, further research is necessary.

This work comprehensively analyzed the FLVCR2 level within AML by referring to several public databases on public bioinformatics platforms. The results showed that FLVCR2 had high expression within AML, which predicted low survival rates. Pathway analysis showed that FLVCR2 was related to TME and immune-related pathways. Moreover, high FLVCR2 expression was related to infiltration degrees of immune cells and immune scores for AML cases, indicating that FLVCR2 possibly had an important effect on AML progression through immune reaction. FLVCR2 silencing suppressed AML cells proliferation and promoted their apoptosis. Our results provide insights into the AML tumorigenesis mechanism and identify FLVCR2 as the significant independent predictor of AML.

## Materials and methods

### Data acquisition

This study obtained transcriptome RNA sequencing (RNA-seq) and relevant clinical data for the tumor and normal samples from The Cancer Genome Atlas (TCGA) database (https://portal.gdc.cancer.gov) and then eliminated cases that had incomplete clinical data. This study gained approval from the Ethics Committee of Qilu Hospital (Jinan, China). TCGA database is publicly available under specific guidelines, and the analysis in this study was conducted following the Declaration of Helsinki. This study also obtained the GSE12417 dataset (Metzeler et al., 2008) from the Gene Expression Omnibus (GEO) database (http://www.ncbi.nlm.nih.gov/geo/) to assess the relation between FLVCR2 expression and prognosis.

### Detection of differential expression of FLVCR2 in AML

This study used the Gene Expression Profilin Interactive Analysis (GEPIA) online database (http://gepia.cancer-pku.cn/index.html) to evaluate the association of FLVCR2 level with clinicopathological features of AML (Tang et al., 2017). Differential gene expression between patients with AML and normal controls was analyzed using the “Expression DIY” module of GEPIA, matching TCGA-derived Genotype-Tissue Expression Project (GTEx) and normal data, as well as log2 (TPM +1). The “Survival” module in GEPIA was used to evaluate the relation between FLVCR2 level and AML survival.

### Gene set enrichment analysis for FLVCR2

This study conducted GSEA based on TCGA-derived RNA-seq data after normalization. GSEA was conducted using the R package “clusterProfiler” (v4.0.3) to identify the potential biological processes and pathways related to FLVCR2 expression in AML progression (Subramanian et al., 2005). This study obtained the HALLMARK gene set from the Molecular Signatures Database. Analyses were repeated 1,000 times. A threshold for significant enrichment was set as a false discovery rate (FDR) < 0.05 and *p* < 0.05.

### Survival analysis of FLVCR2 expression in AML

This study used the online database Kaplan—Meier plotter (www.kmplot.com), including clinical and gene expression profiles (Gyorffy et al., 2013); the TIMER 2.0 online platform (http://timer.comp-genomics.org/) (Li et al., 2020); and PrognoScan (http://dna00.bio.kyutech.ac.jp/PrognoScan/) (Mizuno et al., 2009) to evaluate whether FLVCR2 level predicted AML prognosis. We used an AML dataset to explore the effect of FLVCR2 expression on OS in AML cases. This study also determined hazard ratios and log-rank *p* values. P < 0.05 was the statistical significance.

### Functional annotations

Differentially expressed genes (DEGs) were conducted using Gene Ontology (GO), and Kyoto Encyclopedia of Genes and Genomes (KEGG) analysis with the DAVID database (v6.8; https://david.ncifcrf.gov/summary.jsp) (Huang et al., 2009): *p* < 0.05 was the significance level. The GO enrichment analysis of FLVCR2 revealed biological process (BP), cellular component (CC), and molecular function (MF) terms. Pathways related to FLVCR2 activity along and its co-expression genes were identified by KEGG analysis. The results of enrichment analysis are shown using corrected *p* values and normalized enrichment scores.

### Protein—protein interaction network analysis of FLVCR2

This study applied the database Search Tool for the Retrieval of Interacting Genes/Proteins (STRING) (https://string-preview.org/), used for retrieving interacting genes and PPI networks, to map the FLVCR2 gene and protein interactions. Significant enrichment was assessed by the thresholds of FDR < 0.05 and *p* < 0.01.

### Immune infiltration analysis

“ESTIMATE” in an R package (Yoshihara et al., 2013) was employed to evaluate the stromal and immune scores of tumor samples. We divided samples into two groups according to the median stromal/immune scores. The difference in the survival probability between the two groups was assessed by Cox regression analysis. Additionally, to evaluate the changes in gene levels in samples, the gene level-based deconvolution algorithm CIBERSORT (http://cibersort.stanford.edu/) was used (Bense et al., 2016; Liu et al., 2017). Proportions of 22 immune cell types within AML were examined through CIBERSORT to assess their correlation with survival probabilities. The relation between FLVCR2 level and immunocyte infiltration degrees was analyzed. Additionally, the Wilcoxon rank-sum test was conducted to compare immunocyte infiltration degrees between low- and high-FLVCR2 expression samples. A *p* value of <0.05 was the threshold for selecting lymphocytes that might be affected by FLVCR2 level.

### Patient specimens

This study acquired bone marrow from 30 subjects with AML and 24 normal subjects, both from Qilu Hospital of Shandong University, China. Next, mononuclear cells were isolated using Ficoll—Paque centrifugation. All subjects provided informed consent as stipulated in the Declaration of Helsinki. Each laboratory experiment using primary specimens gained approval from the Medical Ethics Committee of Qilu Hospital of Shandong University.

### Cell culture and transfection

This study obtained human leukemic MOLM-13 and THP-1 cells from the Institute of Hematology and Blood Diseases Hospital, Chinese Academy of Medical Sciences and Peking Union Medical College, Tianjin, China. Next, cells within RPMI-1640 that contained 10% fetal bovine serum were cultivated. FLVCR2 gene sequences were later searched against the National Center for Biotechnology Information’s Gene database. In addition, RNA interference (RNAi) sequences were designed based on the appropriate design principles. GenePharma (Shanghai, China) was responsible for preparing the negative control (NC) and FLVCR2-targeting small interference RNA (siRNA). Lipofectamine RNAiMAX (Invitrogen) was used for transfection within the 6-well plates.

### Quantitative reverse-transcription PCR

This study used TRIzol (Invitrogen) to extract total RNA according to specific protocols and measured it using UV spectrophotometry (DeNovix). Subsequently, an RNA sample (1,000 ng) was collected to prepare cDNA through reverse transcription in 10 μl reaction volume using Evo M-MLV RT Premix for qPCR (AG11706). The SYBR Green Premix Pro Taq HS qPCR Kit (AG11701) was applied in RT-qPCR on the Light Cycler 480 Ⅱ (Roche). The results were calculated by the 2^-ΔΔCT method. GAPDH was used as endogenous control.

### Western blotting assay

M-PERTM Mammalian Protein Extraction Reagent (Thermo Fisher) was used for cell lysis. After separation through SDS-PAGE, proteins were transferred onto nitrocellulose membranes (Millipore). This study used GAPDH as the reference. Anti-FLVCR2 (proteintech) and anti-GAPDH (ZSGB-BIO China) primary antibodies were used. Next, this assay was incubated with HRP Substrate Luminol Reagent (Millipore), and exposure was performed with ChampChemi (SageCreation).

### Cell proliferation test

The proliferation of transfected cells *in vitro* was analyzed by EdU cell proliferation assay. For EdU assays, treated cells were incubated by the iClick™ EdU Andy Fluor™ 647 Flow Cytometry Assay Kit (GeneCopoeia, A008) and tested by flow cytometry (FCM).

### Cell apoptosis analysis

After transfection, supernatants of the cell culture were eliminated before cell collection. After washing with phosphate-buffered saline twice, the cells were added with 1× binding buffer (300 μl). Next, the cells were subject to Annexin V/PI staining (BestBio, Shanghai, China) and FCM (Beckman Coulter) analysis of cell apoptosis within 1 h.

### Statistical analysis

This study used R software (version 3.6.3) for statistical analysis. The relation between FLVCR2 level and clinical features was analyzed by logistic regression analysis. OS was defined as the duration between the diagnosis date and the all-cause mortality date. This study conducted Cox proportional hazards regression to identify OS-related clinical characteristics in patients with AML. *p* < 0.05 (two-sided) denoted statistical significance.

## Results

### FLVCR2 expression is upregulated in AML

**FIGURE 1:**
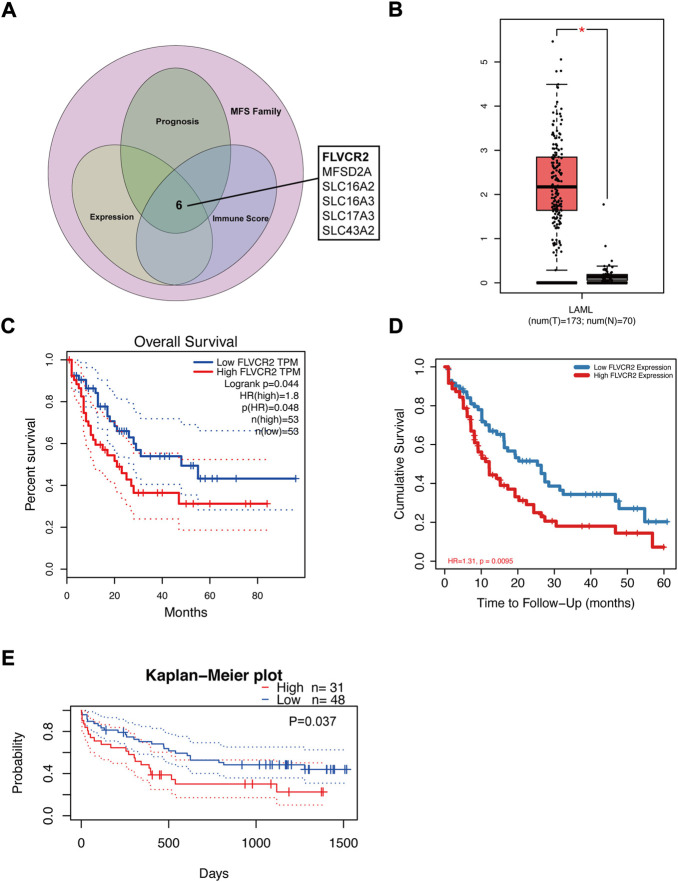
FLVCR2 expression is upregulated in AML and FLVCR2 level correlates with prognosis of AML. **(A)** Schematic graph of screening of MFS Family associated with AML immune score and prognosis. **(B)** The mRNA expression of FLVCR2 in TCGA AML samples (*n* = 173) and the GTEx normal samples (*n* = 70). **(C)** and **(D)** The overall survival analyses of FLVCR2 in AML *via* GEPIA and TCGA online platform. Blue: low FLVCR2 expression; Red: High FLVCR2 expression. **(E)** Comparing the high and low expression levels of FLVCR2 in AML patients with Kaplan—Meier OS curves by PrognoScan (GSE12417-GPL570) *p < 0.05; **p < 0.01, ***p < 0.001.

For the exploration of key genes within AML, DEGs were analyzed in the TCGA dataset, and the significance of these genes was tested using immune scores and AML prognosis. FLVCR2 was one of the six overlapping genes among the MFS members (Figure 1A). The other five were MFSD2A, SLC16A2, SLC16A3, SLC17A3, and SLC43A2 (*p* < 0.05, |log2FoldChange| > 1). We focused on the function of FLVCR2 in the tumorigenesis of AML, and FLVCR2 showed upregulation within AML (Figure 1B). Furthermore, the relation between FLVCR2 and patient clinical features, including race, gender, age, treatment, and neoadjuvant, was investigated (Table 1). The FLVCR2 level showed a strong relation to neoadjuvant and treatment (*p* < 0.05). Additionally, the proportion of AML cases with low FLVCR2 expression was higher than that with high FLVCR2 expression without treatment or neoadjuvant. However, no other differences were observed between low- and high-FLVCR2 expression groups.

**TABLE 1:** Description of clinical characteristics for 151 AML sample in both FLVCR2 high/low groups from TCGA.

Clinical characteristics	FLVCR2—high (*n* = 76)	FLVCR2—low (*n* = 75)	Total (*n* = 151)	*p* Value
Age	57.18 (16.79)	53.13 (15.35)	54.17 (16.07)	0.434
Gender				
Male	40 (52.63)	43 (57.33)	83 (54.97)	0.677
Female	36 (47.37)	32 (42.67)	68 (45.03)	
Race				
White	69 (90.79)	66 (88)	135 (89.40)	0.625
Asian	1 (1.32)	0 (0.00)	1 (0.66)	
Black or African American	5 (6.58)	8 (10.67)	13 (8.61)	
Not reported	1 (1.32)	1 (1.33)	2 (1.32)	
Neoadjuvant				
Yes	25 (32.89)	13 (17.33)	38 (25.17)	0.044
No	51 (67.11)	62 (82.67)	113 (74.83)	
Prior malignancy				
Yes	8 (10.53)	5 (6.67)	13 (8.61)	0.579
No	68 (89.47)	70 (93.33)	138 (91.39)	
Prior treatment				
Yes	25 (32.89)	13 (17.33)	38 (25.17)	0.044
No	51 (67.11)	62 (82.67)	113 (74.83)	

*Data are mean ± SD, and frequency (percent) for numeric and category variables, respectively.

### FLVCR2 level correlates with prognosis of AML

To further understand FLVCR2’s effect on AML, its prognostic role in AML was assessed using the GEPIA database and RNA-seq data. The results showed that high FLVCR2 expression levels predicted poor OS of the patients with AML (Figure 1C). Furthermore, we used TCGA database to assess the correlation with FLVCR2 level and FLVCR2’s prognostic significance for AML. According to the results, FLVCR2 expression in patients with AML was higher than that in normal controls. According to the median expression level of FLVCR2, we divided the AML patients into high FLVCR2 expression group (*n* = 76) and low FLVCR2 expression group (*n* = 75). We found that the low FLVCR2 expression was correlated with superior OS in the median cutoff groups in AML (Figure 1D). We also studied the relation between FLVCR2 level and prognostic outcome by using the GSE12417-GPL570 dataset (Figure 1E). Comparing the high and low expression levels of FLVCR2 in AML patients with Kaplan–Meier OS curves by PrognoScan, high FLVCR2 expression levels were also corresponded with poor OS prognosis in AML (Figure 1E). Based on the aforementioned findings, FLVCR2 level was significantly related to AML prognosis and progression.

### Protein—protein interaction network and functional annotation of FLVCR2 in AML

**FIGURE 2:**
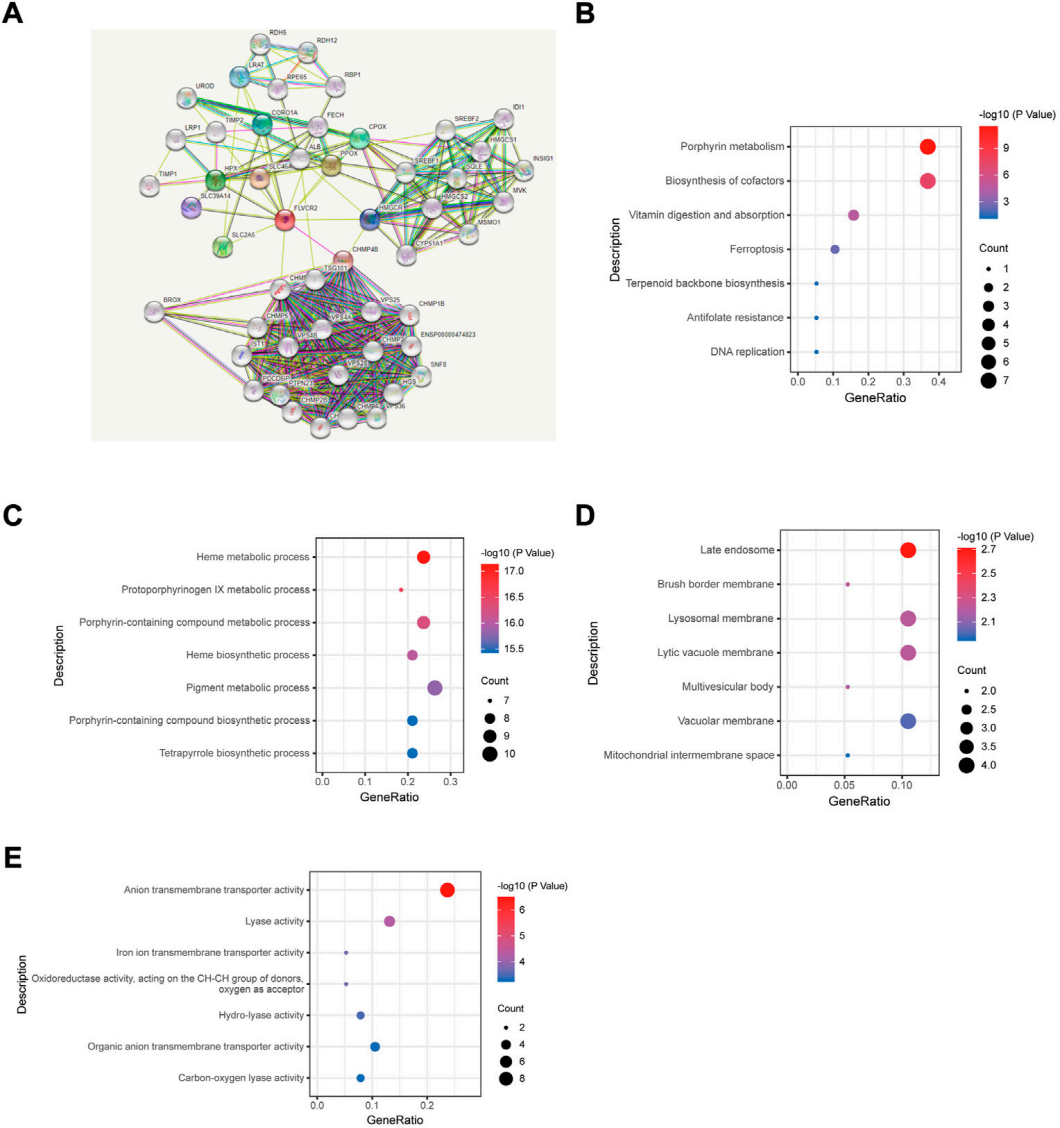
Protein—protein interaction network and functional annotation of FLVCR2 in AML. **(A)** Integrated protein-protein interaction network of FLVCR2 *via* STRING. **(B)** Top seven enriched KEGG pathway distribution. **(C–E)** GO enrichment analysis result of cellular component, molecular function, and biological process.

Next, we constructed a protein—protein interaction network based on DEGs by using the STRING database to screen for FLVCR2-binding proteins. The results showed that a subset of immune-related proteins interacted with FLVCR2, including HMGCR and CORO1A (Figure 2A). In AML, HMGCR and CORO1A showed a significant correlation with FLVCR2. Studies have showed that HMGCR and CORO1A were involved in regulating T-cell homeostasis, which was important for the tumor microenvironment. These findings made us interested in the role of FLVCR2 in immunity in AML. FLVCR2 might affect the immunomodulation and progression of AML.

To understand FLVCR2’s functional implications within AML between two groups, we performed GO and KEGG pathway enrichment analyses. By means of GO enrichment analysis, we filtered the top seven cellular components (CC), biological process (BP), and molecular function (MF) categories based on the following criteria: *p* value <0.01 and FDR <0.05 (Figures 2C–E). The GO-BP terms involved in the metabolic process were discovered. Additionally, GO-CC terms were related to transporter activity. Moreover, KEGG pathway analysis (Figure 2B) indicated the enrichment of metabolism, ferroptosis, and DNA replication pathways, which were key pathways closely related to tumorigenesis. This may explain the correlation between the FLVCR2 higher expression level and poor prognosis in AML.

### FLVCR2 regulates immune response in AML

**FIGURE 3:**
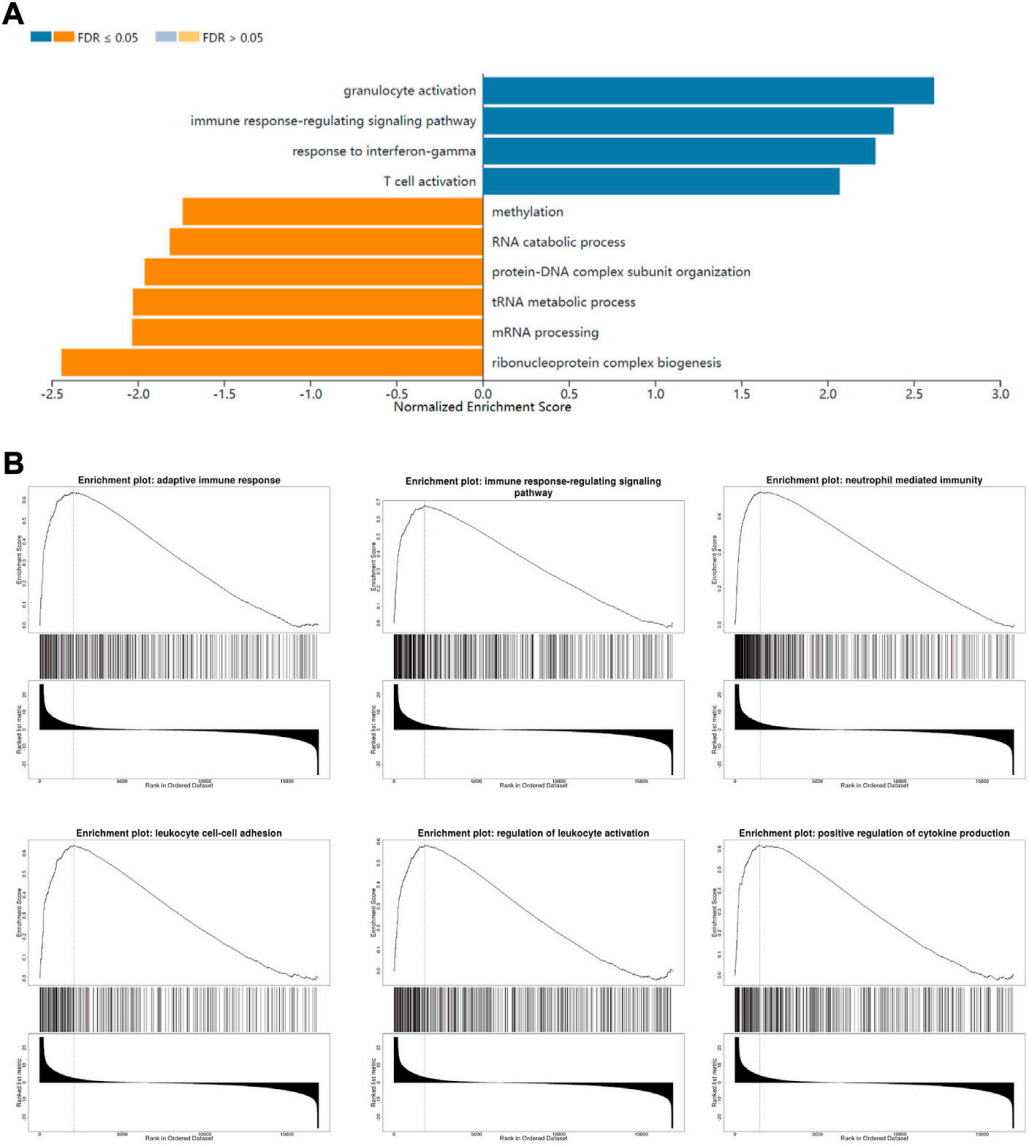
FLVCR2 regulates immune response in AML. **(A)** Significantly enriched biological processes of GSEA annotations of FLVCR2 in AML (LinkedOmics). **(B)** GSEA enrichment plots of specified biological processes.

To understand FLVCR2’s functional implications within AML between two groups, we conducted GSEA for FLVCR2, based on LinkedOmics database, to investigate the possible BPs and pathways. Based on the normalized enrichment scores, the most significantly enriched signaling pathway in terms of FLVCR2 gene expression was selected. As shown in Figures 3A,B, high levels of FLVCR2 were enriched in immunoactivity-related pathways, including the immune response signaling pathway, intercellular adhesion of leukocytes, leukocyte activation regulation, positive cytokine production regulation, and neutrophil-mediated immunity. The analysis also revealed categories negatively correlated with high FLVCR2 levels: methylation, RNA catabolic processing, mRNA processing, tRNA metabolic process, protein-DNA complex subunit organization, and ribonucleoprotein complex biogenesis. These results prompted us to conduct a subsequent study on the effect of FLVCR2 on immune infiltration in AML.

### Relation between FLVCR2 expression and TME

**FIGURE 4:**
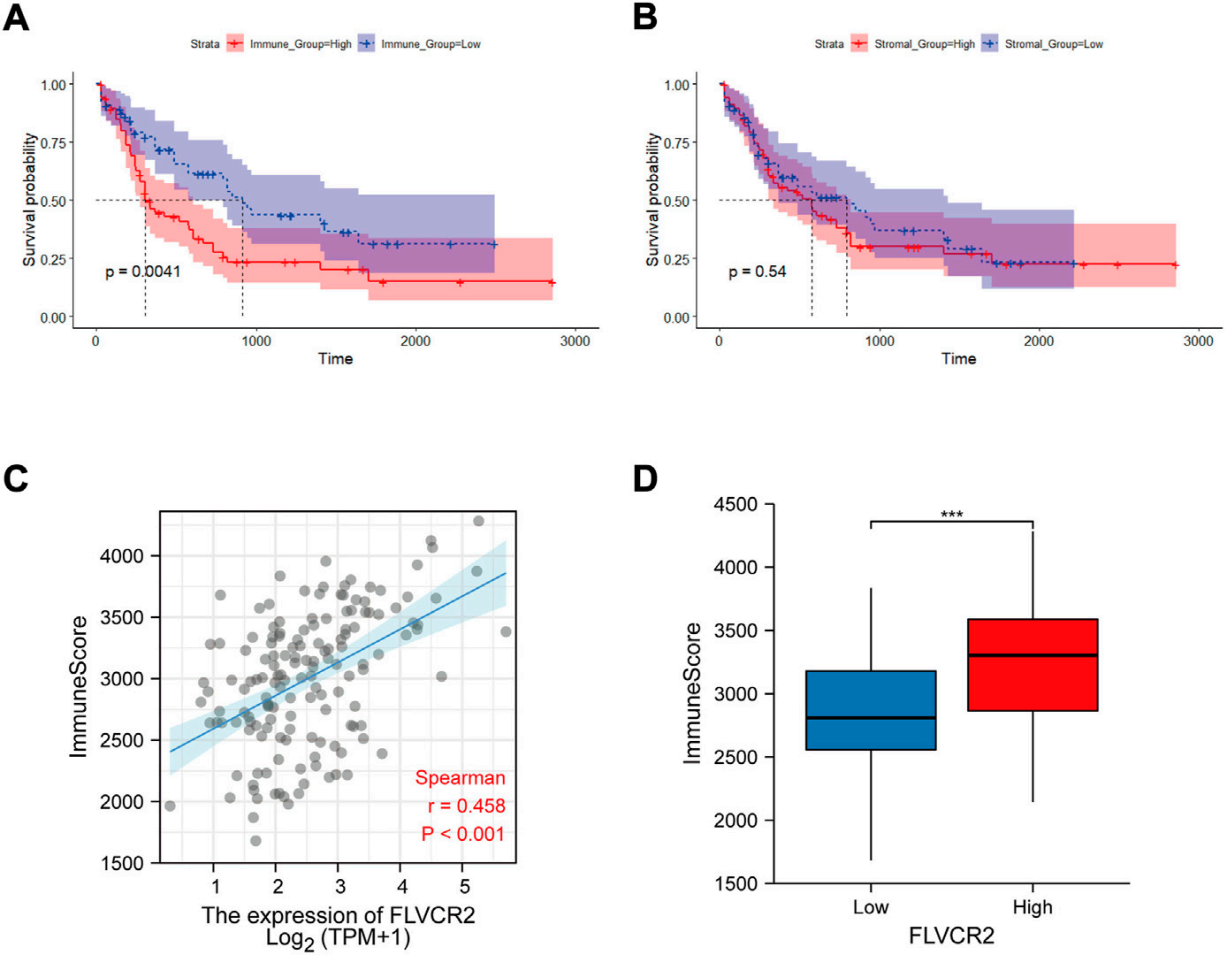
Relation between FLVCR2 expression and tumor immune environment. **(A,B)** Correlation analysis between OS and immune score (left) or stromal score (right). **(C,D)** Correlation between FLVCR2 and immune score by linear graph (left) or dichotomy graph (right). *p < 0.05; **p < 0.01, ***p < 0.001.

FLVCR2 level has been suggested to be associated with immune pathways in AML. Because immune and stromal cells represent the main TME components, this study analyzed the effect of FLVCR2 on TME. First, the stromal and immune scores of AML cases were determined. Next, the effects on the prognostic outcome were assessed. The results showed that low immune scores predicted improved prognosis, but stromal scores were not significantly related to OS (Figures 4A,B). Further correlation analysis showed that FLVCR2 expression showed a positive relation to immune score, with significant difference detected through dichotomization (Figures 4C,D). This result suggests the role of FLVCR2 in affecting TME activity and immunocyte infiltration.

### FLVCR2 level relates to immunocyte infiltration in AML

**FIGURE 5:**
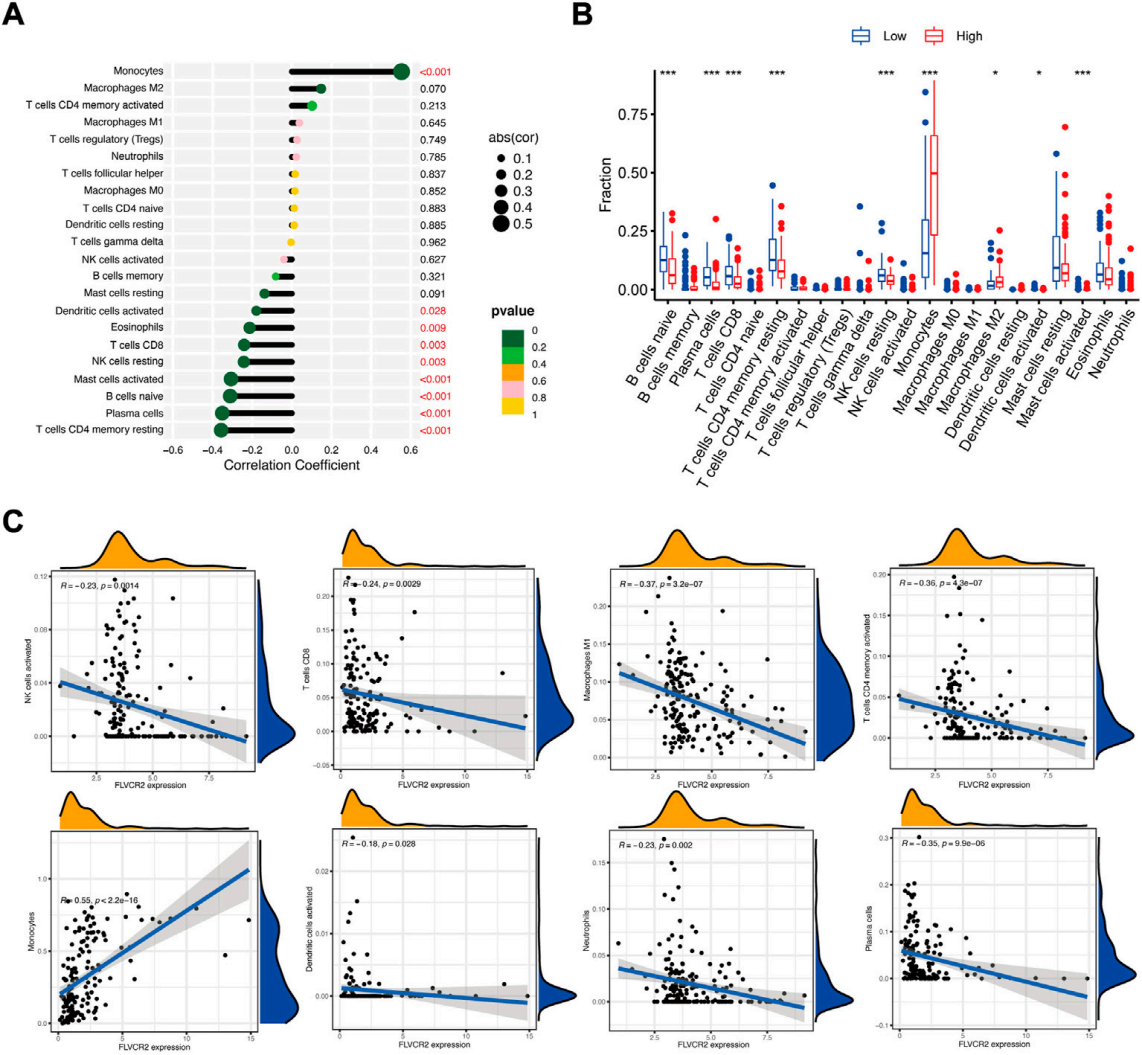
FLVCR2 level relates to immunocyte infiltration in AML. **(A)** FLVCR2 is correlated with immune infiltration in AML. **(B,C)** Correlations between FLVCR2 expression and the infiltration abundances of selected immune cells in AML through CIBERSORT algorithm. *p < 0.05; **p < 0.01, ***p < 0.001.

Next, we examined the association between FLVCR2 mRNA level and immunocyte infiltration. According to correlation analysis, FLVCR2 expression showed a positive relation to the infiltration degrees of monocytes and macrophages M2 (Figure 5A). Furthermore, this study used the CIBERSORT algorithm to explore gene levels in the samples to calculate scores for 22 immunocyte subtypes between low and high FLVCR2 expression groups (Figure 5B). As shown in Figure 5B, plasma cells, naive B cells, CD8^+^ T cells, resting NK cells, CD4^+^ memory T cells, monocytes, mast cells, and M2 macrophages represented the main immune cells affected by FLVCR2 level. For AML cases, FLVCR2 upregulation was significantly related to the high proportion of monocytes. However, FLVCR2 upregulation was negatively associated with some immunocyte types, such as plasma cells, activated CD4 T cells memory, activated NK cells, CD8^+^ T cells, M1 macrophages, and activated dendritic cells (Figure 5C). The results showed that FLVCR2 expression was significantly related to the immune infiltration level, which might contribute to an immune inhibitory phenotype of TME in AML. These findings led a heavy hint that FLVCR2 affects patient survival *via* interacting with immune infiltration in AML.

### FLVCR2 differential analysis among several cancers

**FIGURE 6:**
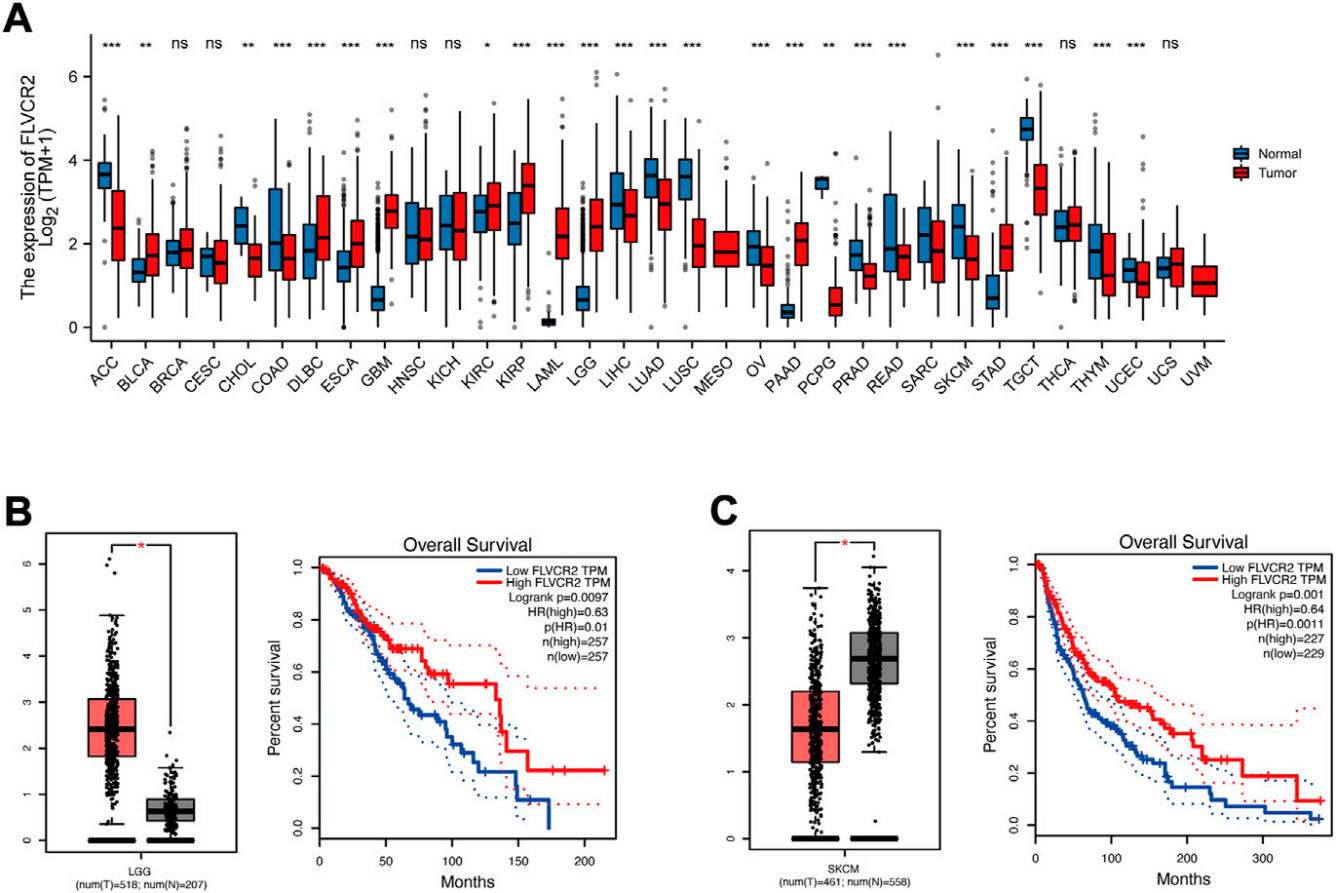
FLVCR2 differential analysis among several cancers. **(A)** The mRNA expression of the FLVCR2 expression profile across multiple tumor samples (red) and paired normal tissues (blue). **(B)** The mRNA expression of FLVCR2 in LGG samples (tumor samples in red from TCGA, and normal samples in gray from GTEx). The overall survival analyses of FLVCR2 in LGG. Blue: low FLVCR2 expression; Red: High FLVCR2 expression. **(C)** The mRNA expression of FLVCR2 in SKCM samples (tumor samples in red from TCGA, and normal samples in gray from GTEx). The overall survival analyses of FLVCR2 in SKCM. Blue: low FLVCR2 expression; Red: High FLVCR2 expression. *p < 0.05; **p < 0.01, ***p < 0.001.

For exploring FLVCR2 expression between cancer and healthy samples from several cancers, this study used web server TCGA and GTEx. FLVCR2 expression increased in tumors, such as renal papillary cell carcinoma, renal clear cell carcinoma, and low-grade glioma (LGG), compared to that in matched healthy samples (Figure 6A), and FLVCR2 downregulation predicted the dismal OS for 518 LGG cases (Figure 6B). However, the expression level of FLVCR2 in skin cutaneous melanoma (SKCM) decreased relative to that in healthy samples, and low FLVCR2 expression predicted poor SKCM patient survival (Figure 6C).

### FLVCR2 shows high expression in primary AML specimens

**FIGURE 7:**
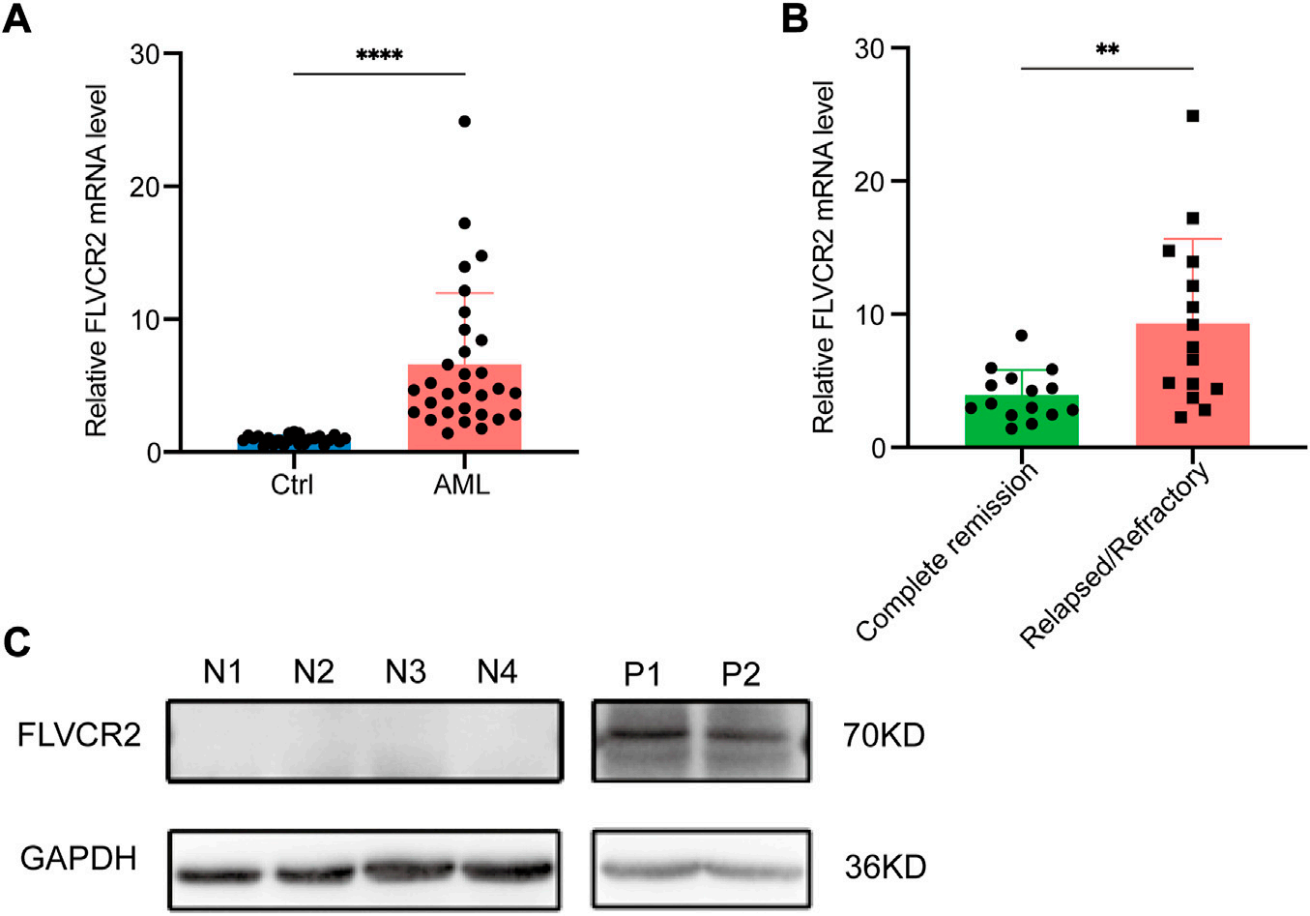
FLVCR2 shows high expression within primary AML specimens. **(A)** Quantitative mRNA expression of FLVCR2 by RT-qPCR in AML patient samples (n = 30) and controls (Ctrl, *n* = 24). **(B)** Quantitative mRNA expression of FLVCR2 in patients with relapsed or refractory AML (*n* = 15) and patients with complete remission (*n* = 15). *p < 0.05; **p < 0.01, ***p < 0.001. **(C)** Representative western blotting analysis of FLVCR2 protein level in patients with newly-diagnosed AML (P1 and P2, *n* = 2) relative to control (N1-N4, *n* = 4). In A and B, *p* values were determined by two-sided unpaired *t*-test.

This study measured FLVCR2 mRNA expression in bone marrow specimens collected from AML cases (*n* = 30) and healthy subjects (*n* = 24). Table 2 displays clinical features. The FLVCR2 level significantly elevated in AML bone marrow in comparison with that of controls (*p* < 0.01, Figure 7A). The expression of FLVCR2 depended on the efficacy of the therapy. The FLVCR2 levels were significantly reduced during therapy when patients achieved complete remission but remained constant in those with no remission. FLVCR2 expression was higher in relapsed or refractory AML cases (*n* = 15, *p* < 0.01) than in those achieving complete response (*n* = 15, Figure 7B).

**TABLE 2:** Clinical characteristics of AML patients.

Clinical characteristics	AML patients
Median age (range), years	55 (30–85)
Male,n	17
Female,n	13
Blast in bone marrow,%	
Median	44.82
Range	10.19–94
FAB classification	
M0	1
M1	1
M2	3
M3	3
M4	4
M5	17
M6	1
M7	0
WBC, × 10^9^/L	
Median	10
Range	0.98–172.61
Gene Mutation	
FLT3-ITD	6
CEBPA	7
IDH1/2	5
AML1	4
Gene fusion	
PML-RARa	3
AML1-ETO	3

Note: AML, acute myeloid leukemia; WBC, white bloodcells; FAB French-American-British classification.

Furthermore, we detected the FLVCR2 protein expression levels, and confirmed that the protein level of FLVCR2 was increased in AML patients compared with that in healthy donors (Figure 7C). Based on the aforementioned results, FLVCR2 level was associated with treatment effect.

### FLVCR2 inhibition suppresses AML cells proliferation and promotes their apoptosis

**FIGURE 8:**
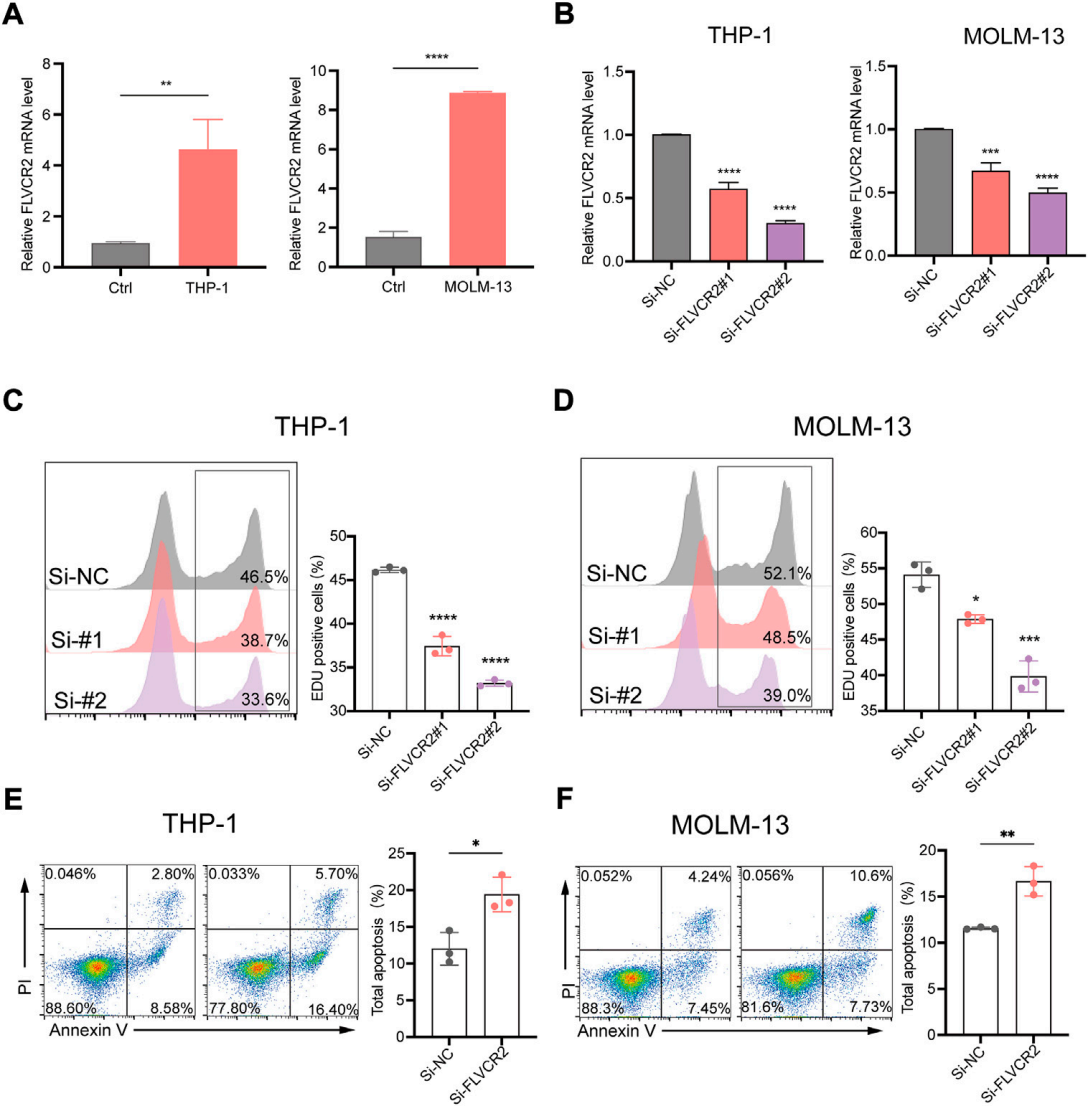
FLVCR2 inhibition suppresses AML cell proliferation and promotes their apoptosis. **(A)** Quantitative mRNA expression of FLVCR2 by RT-qPCR in THP-1, MOLM-13 cells and controls. **(B)** THP-1 and MOLM-13 cells were transfected with si-NC, si-FLVCR2#1, or si-FLVCR2#2. Knockdown efficiency of FLVCR2 in cells was measured by RT-qPCR. **(C,D)** Cell proliferation was evaluated by EdU assay, and si-FLVCR2 inhibited THP-1 and MOLM-13 cell proliferative activities compared with the si-NC group. **(E,F)** After 48 h of transfection, cell apoptosis was measured by flow cytometry. The cell apoptotic rates between si-NC and si-FLVCR2 groups were analyzed in THP-1 and MOLM-13 cells. N = 3.*p < 0.05; **p < 0.01, ***p < 0.001. In B-D, *p* values were determined by one-way ANOVA; In Aand E-F, *p* values were determined by two-sided unpaired *t*-test.

To improve the exploration of FLVCR2’s function during AML progression, we prepared siRNAs targeting FLVCR2 (si-FLVCR2) and NC siRNAs (si-NC). The FLVCR2 level markedly elevated within THP-1and MOLM-13 cells in comparison with that of heathy controls (*p* < 0.01, Figure 8A). Next, we examined siRNA knockdown efficiency. The results showed that transfection with si-FLVCR2 significantly decreased the FLVCR2 level within THP-1 and MOLM-13 cells relative to si-NC (Figure 8B). The proliferation of THP-1 and MOLM-13 cells was examined using the EdU proliferation assay. The results showed that si-FLVCR2 transfection markedly suppressed the proliferation of THP-1 and MOLM-13 cells (*p* < 0.05, Figures 8C,D). FCM was conducted to analyze THP-1 and MOLM-13 cell apoptosis after FLVCR2 interference. The results showed that FLVCR2 interference promoted THP-1 and MOLM-13 cell apoptosis (Figures 8E,F). In conclusion, FLVCR2 has a critical effect in regulating cell apoptosis and maintaining cell proliferation in AML.

## Discussion

AML is a highly heterogeneous, malignant tumor with varied prognoses. The pathogenic mechanisms underlying AML have not been fully elucidated. Therefore, novel biomarkers are necessary to understand mechanisms related to AML. FLVCR2 was reported to be associated with Fowler syndrome (Meyer et al., 2010). Subsequently, FLVCR2 was found to be a potential biomarker for predicting prostate cancer (Alkhateeb et al., 2019). Nonetheless, its effect on AML remains unclear. In this study, FLVCR2 level was considered an essential predictor of clinical outcomes of AML.

The study examined FLVCR2’s level, prognostic significance, and potential mechanism in AML by using TCGA datasets. The results showed that FLVCR2 mRNA levels increased more in AML samples than in normal samples according to the results obtained through GEPIA and GEO database. FLVCR2 level elevated in relapsed/refractory AML cases relative to complete remission AML cases, suggesting that FLVCR2 level might be related to treatment efficacy and prognostic value. We then performed prognostic analysis and found that FLVCR2 upregulation was associated with poor prognosis, suggesting that FLVCR2 functions as an oncogene. DEGs associated with increased FLVCR2 levels were enriched in immunity-associated pathways. Our findings indicate that FLVCR2 may be an independent prognostic factor that influences tumorigenesis and tumor immunology in AML progression. However, its biological involvement in AML remains to be explored.

Furthermore, we investigated the biological functions and mechanisms of action of FLVCR2 in AML. The literature (Pao et al., 1998) showed that FLVCR2 was an MFS transporting small substances across the membranes upon chemiosmotic gradient stimulation, which helped to maintain homeostasis in cells. In our study, the functional annotation of FLVCR2 revealed that cell–cell adhesion, cellular defense response, translational initiation, and ribonucleoprotein complex biogenesis were closely associated with FLVCR2 expression. Studies have shown that adhesion molecules are closely related to tumorigenesis and development (Sisto et al., 2021; Ito et al., 2022), which is consistent with our results. The cell–cell adhesion events enriched in the high FLVCR2 expression group show that FLVCR2 plays a critical role in AML tumorigenesis. Therefore, although the precise mechanisms of tumor progression have not been elucidated, the aforementioned signaling pathways were confirmed to be related to AML.

Moreover, PPI network analysis revealed that CORO1A and HMGCR had a low but significant correlation with FLVCR2. CORO1A, responsible for encoding the Trp-Asp repeat protein, is related to different cell events. CORO1A depletion possibly results in compromised T-cell activity and homeostasis due to the defect in the T-cell receptor pathway (Mueller et al., 2008). Additionally, HMGCR primarily regulates the activation, survival, progression, and resistance of T lymphocytes. The changed HMGCR level regulates immune function, and the metabolism status of T cells. HMGCR synthetase represents the enzyme involved in purine biosynthesis pathway, which is related to increased cancer phenotypes such as tumor cell invasion and growth. These findings further highlight that FLVCR2 has an important effect on the genesis and immunomodulation of AML.

Immune-based therapies have become important in cancer treatment (Abril-Rodriguez and Ribas, 2017). However, patients receiving immunotherapy finally experience drug resistance because of immune escape. TME has gained attention in mediating immune reactions in cancer, and targeting TME components is expected to be a novel treatment strategy for AML. Studies have verified that the interactions between TME and AML are mainly mediated by various immune cells, which have critical effects on cancer immune escape (Li et al., 2020). TME is mainly composed of myeloid-derived suppressor cells, macrophages, and tumor-infiltrating lymphocytes (TILs) (Mitteldorf et al., 2017). TILs represent a key part of the TME and are classified into two types: tumor-antagonizing and tumor-promoting immune cells (Lei et al., 2020). Studies have found that TME contributes to immunological changes during AML progression (Tettamanti et al., 2022). Therefore, this study analyzed the relation between FLVCR2 and immune infiltration within AML. According to our review of the literature, this study is the first to support the link between FLVCR2 and tumor immunity in AML. We found that high FLVCR2 expression showed a negative relation to NK cells, CD4^+^ T cells, and CD8^+^ T cells, whereas a positive relation was observed with Tregs and M2 macrophages in AML. Tumor eradication by CD8^+^ T cells involves tumor antigens, antigen presentation, T-cell activation, and tumor cell killing (Steven et al., 2016). The low NK cell and CD8^+^ T cell proportions within tumors predict a dismal AML prognosis (Zhou et al., 2011; Truxova et al., 2020). By contrast, some articles have suggested that a high tumor-infiltrating Tregs proportion predicts a poor outcome of AML (Shenghui et al., 2011). Therefore, upregulated FLVCR2 probably suppresses tumor immunity, which contributes to the escape of tumor cells and promotes carcinogenesis, indicating a possible mechanism by which FLVCR2 affects the OS of patients with AML. The *in vitro* results demonstrated that FLVCR2 interference inhibited cell proliferation activities and increased the incidence of apoptosis, verifying that FLVCR2 was important for regulating cell apoptosis and maintaining cell proliferation within AML.

Previous studies have confirmed the importance of identifying a biomarker for tumorigenesis and development of particular cancer by combining bioinformatics analysis and experimental demonstration. Gao et al. (2021) found that SLC2A3 was correlated with EMT and immune signature in colorectal cancer. Fan et al. (2022) verified that SLC25A38 was a novel biomarker for metastasis and clinical outcome in uveal melanoma. Our study focused on identifying FLVCR2 as a novel biomarker for AML, which may be an independent prognostic factor that influenced tumorigenesis and tumor immunology in AML progression. Despite our findings, this study has limitations. Hence, further investigations should analyze mechanisms related to FLVCR2-medicated tumor immunity and biological functions *in vivo*.

In conclusion, this study first identifies the prognostic value of FLVCR2 in patients with AML. FLVCR2 expression is associated with immunocyte infiltration, such as NK cells, T cells, or macrophages in AML. Our results suggest that FLVCR2 may predict treatment outcomes and prognosis in patients with AML and play a critical role in regulating cell apoptosis and maintaining cell proliferation within AML. With an improved understanding of its function, FLVCR2 may be developed as a component of targeted therapies for AML treatment.

## Data Availability

The datasets presented in this study can be found in online repositories. The names of the repository/repositories and accession number(s) can be found in the article/Supplementary Material.

## Abbreviations

AML, Acute myeloid leukemia; BP, Biological process; CC, Cellular component; DEGs, Differentially expressed genes; FCM, Flow cytometry; GEO, Gene Expression Omnibus; GEPIA, Gene Expression Profiling Interactive Analysis; GO, Gene Ontology; GSEA, Gene set enrichment analysis; KEGG, Kyoto encyclopedia of genes and genomes; LGG, Low-grade glioma; MF, Molecular function; MFS, Major facilitator superfamily; NC, Negative control; NK, Natural killer; OS, Overall survival; PPI, Protein–protein interaction; SKCM, Skin cutaneous melanoma; TCGA, The Cancer Genome Atlas; TILs, Tumor-infiltrating lymphocytes; TME, Tumor microenvironment; Tregs, regulatory T cells.
