# Supplementary figures and images for: The prognostic marker FLVCR2 associated with tumor progression and immune infiltration for acute myeloid leukemia
Source: Front Cell Dev Biol. 2022 Oct 12;10:978786. doi: 10.3389/fcell.2022.978786 (PMC9597318; doi:10.3389/fcell.2022.978786)

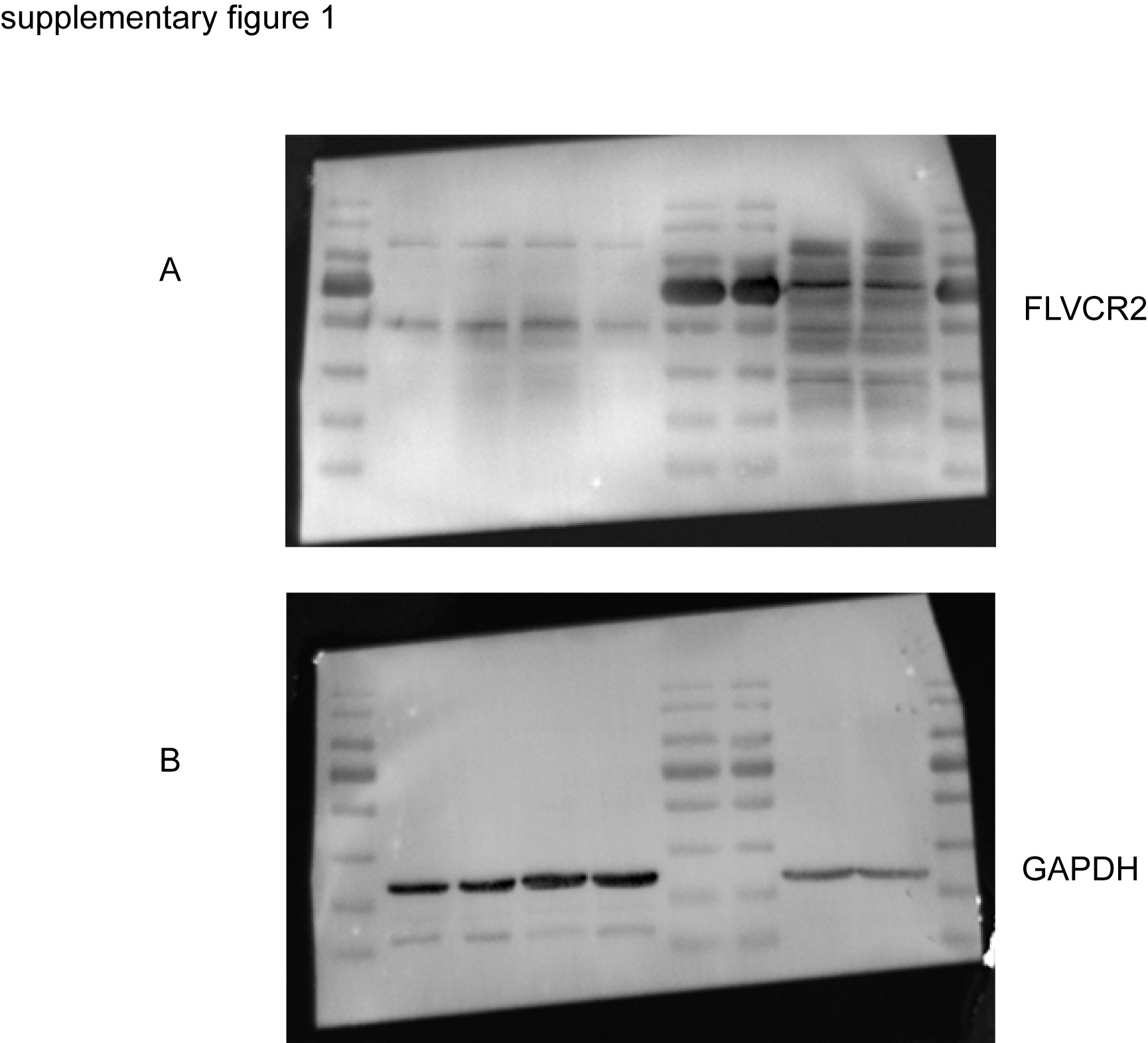

Supplement: Supplementary file 1 [file Image1.JPEG]
