# Supplementary material for: The prognostic marker FLVCR2 associated with tumor progression and immune infiltration for acute myeloid leukemia
Source: Front Cell Dev Biol. 2022 Oct 12;10:978786. doi: 10.3389/fcell.2022.978786 (PMC9597318; doi:10.3389/fcell.2022.978786)
Supplement: Supplementary file 2 [file DataSheet1.DOCX]

**Supplementary Table 1** Raw data for Figure 1

| Genes | High_ImmuneScore | Low_ImmuneScore | baseMean | log2FoldChange | *P-Immu* | HR (95% CI for HR) | *P-Prog* |
| --- | --- | --- | --- | --- | --- | --- | --- |
| MFSD3 | 322.071 | 416.951 | 369.197 | -0.373 | 0.022 | 1.2 (0.89-1.7) | 0.21 |
| SLC2A1 | 2113.194 | 2003.492 | 2058.706 | 0.077 | 0.763 | 0.94 (0.73-1.2) | 0.67 |
| SLC18A2 | 258.568 | 488.118 | 372.583 | -0.917 | 0.012 | 0.97 (0.79-1.2) | 0.81 |
| SLC43A2 | 2846.895 | 579.716 | 1720.813 | 2.296 | <0.001 | 1.3 (1-1.5) | 0.018 |
| SPNS1 | 282.865 | 239.908 | 261.529 | 0.238 | 0.03 | 1 (0.54-1.9) | 0.96 |
| SLC37A3 | 354.073 | 229.707 | 292.302 | 0.624 | 0.005 | 0.94 (0.63-1.4) | 0.77 |
| SLC22A15 | 1458.502 | 1327.046 | 1393.209 | 0.136 | 0.76 | 0.99 (0.87-1.1) | 0.89 |
| MFSD6L | 66.742 | 85.228 | 75.924 | -0.352 | 0.077 | 0.79 (0.51-1.2) | 0.29 |
| SLC22A12 | 17.564 | 45.635 | 31.506 | -1.376 | <0.001 | 1 (0.64-1.6) | 0.94 |
| SLC33A1 | 1345.412 | 1400.849 | 1372.947 | -0.058 | 0.407 | 0.47 (0.22-1) | 0.05 |
| SLC37A4 | 849.83 | 1047.319 | 947.92 | -0.302 | 0.007 | 1 (0.66-1.6) | 0.86 |
| SLC22A2 | 0.796 | 0.585 | 0.691 | 0.388 | 0.438 | 81 (1.2e-09-5.3e+12) | 0.73 |
| SPNS2 | 1591.642 | 3061.985 | 2321.945 | -0.944 | <0.001 | 0.94 (0.8-1.1) | 0.47 |
| SLC2A4 | 47.397 | 44.569 | 45.993 | 0.088 | 0.824 | 0.75 (0.4-1.4) | 0.38 |
| SLC22A9 | 1.276 | 2.363 | 1.816 | -0.917 | 0.017 | 1.8 (0.047-72) | 0.75 |
| MFSD5 | 687.754 | 553.757 | 621.199 | 0.313 | <0.001 | 2.2 (1.3-3.9) | 0.006 |
| SLC2A13 | 312.235 | 309.531 | 310.892 | 0.013 | 0.961 | 0.78 (0.47-1.3) | 0.31 |
| SLC22A5 | 624.761 | 713.001 | 668.589 | -0.191 | 0.331 | 1.3 (0.92-1.8) | 0.14 |
| SLC22A23 | 343.912 | 419.99 | 381.699 | -0.288 | 0.147 | 0.78 (0.52-1.2) | 0.25 |
| SLC16A13 | 186.534 | 132.469 | 159.681 | 0.494 | 0.001 | 1.6 (1.1-2.4) | 0.022 |
| SLC16A4 | 244.689 | 283.729 | 264.08 | -0.214 | 0.055 | 0.69 (0.43-1.1) | 0.14 |
| SLC22A11 | 8.485 | 17.407 | 12.916 | -1.035 | 0.002 | 0.47 (0.091-2.4) | 0.36 |
| SLC2A3 | 6584.347 | 3702.085 | 5152.76 | 0.831 | <0.001 | 0.9 (0.75-1.1) | 0.28 |
| MFSD6 | 1717.493 | 1571.655 | 1645.057 | 0.128 | 0.352 | 1.3 (0.93-1.9) | 0.12 |
| SLC16A7 | 1658.117 | 1791.181 | 1724.208 | -0.111 | 0.502 | 0.73 (0.5-1.1) | 0.098 |
| SLC16A10 | 63.228 | 126.813 | 94.81 | -1.005 | <0.001 | 0.86 (0.46-1.6) | 0.65 |
| SLC22A25 | 0.711 | 0.989 | 0.849 | -0.54 | 0.231 | 5.7 (1.2e-05-2800000) | 0.79 |
| SLC2A11 | 424.128 | 511.398 | 467.474 | -0.27 | 0.005 | 0.99 (0.55-1.8) | 0.97 |
| SLC22A18 | 525.443 | 273.418 | 400.265 | 0.943 | <0.001 | 1.3 (0.91-1.8) | 0.15 |
| SLC37A1 | 1616.721 | 1914.322 | 1764.536 | -0.244 | 0.129 | 1.4 (1-1.8) | 0.039 |
| SLC18A3 | 0.889 | 0.789 | 0.839 | 0.158 | 0.729 | 4.2e-06 (3.6e-11-0.49) | 0.037 |
| MFSD10 | 4651.375 | 5181.202 | 4914.534 | -0.156 | 0.481 | 0.94 (0.76-1.2) | 0.61 |
| SLC16A9 | 129.106 | 256.833 | 192.547 | -0.992 | 0.008 | 0.88 (0.7-1.1) | 0.25 |
| UNC93A | 0.941 | 1.203 | 1.071 | -1.225 | 0.043 | 0.00026 (4.2e-08-1.6) | 0.063 |
| SLC16A2 | 34.37 | 126.96 | 80.358 | -1.884 | <0.001 | 0.63 (0.43-0.93) | 0.02 |
| SLC2A7 | 2.968 | 5.987 | 4.468 | -1.019 | 0.02 | 0.75 (0.26-2.2) | 0.61 |
| MFSD1 | 6399.31 | 4998.728 | 5703.657 | 0.356 | 0.001 | 0.95 (0.61-1.5) | 0.83 |
| SLC18A1 | 12.605 | 9.799 | 11.211 | 0.366 | 0.189 | 0.68 (0.11-4.3) | 0.68 |
| SLC43A1 | 1391.254 | 1882.92 | 1635.459 | -0.437 | <0.001 | 0.77 (0.54-1.1) | 0.15 |
| SLC17A7 | 6.556 | 11.381 | 8.953 | -0.798 | <0.001 | 0.022 (0.00058-0.85) | 0.041 |
| SLC37A2 | 1095.561 | 179.82 | 640.723 | 2.607 | <0.001 | 1.1 (0.94-1.4) | 0.2 |
| SLC17A9 | 4565.018 | 6373.394 | 5463.218 | -0.481 | 0.005 | 0.91 (0.72-1.1) | 0.41 |
| SLC16A5 | 1243.877 | 918.79 | 1082.41 | 0.437 | <0.001 | 1.1 (0.76-1.7) | 0.54 |
| SLC43A3 | 2670.376 | 2167.648 | 2420.676 | 0.301 | 0.014 | 1.4 (0.96-2.2) | 0.077 |
| SLC46A2 | 130.505 | 22.719 | 76.969 | 2.524 | <0.001 | 1.1 (0.82-1.5) | 0.55 |
| SPNS3 | 2462.375 | 2849.338 | 2654.575 | -0.211 | 0.487 | 1.1 (0.97-1.4) | 0.12 |
| MFSD2B | 352.647 | 433.653 | 392.882 | -0.298 | 0.318 | 0.76 (0.59-0.98) | 0.032 |
| SLC17A1 | 0.82 | 1.312 | 1.064 | -0.656 | 0.244 | 21 (0.25-1800) | 0.18 |
| SLC22A3 | 31.214 | 35.101 | 33.145 | -0.169 | 0.746 | 0.91 (0.56-1.5) | 0.72 |
| SLC16A6 | 174.961 | 40.106 | 107.98 | 2.124 | <0.001 | 1 (0.72-1.4) | 0.97 |
| SV2C | 31.645 | 27.99 | 29.83 | 0.175 | 0.525 | 0.37 (0.044-3.1) | 0.36 |
| SVOPL | 57.563 | 91.654 | 74.496 | -0.671 | 0.013 | 0.59 (0.3-1.1) | 0.12 |
| SLC17A4 | 0.674 | 0.611 | 0.642 | 0.107 | 0.867 | 280 (9.2e-07-8.3e+10) | 0.57 |
| MFSD2A | 434.575 | 196.171 | 316.162 | 1.147 | <0.001 | 1.4 (1.1-1.9) | 0.007 |
| SLC22A6 | 0.44 | 0.583 | 0.511 | -0.39 | 0.426 | 2.5 (4.2e-06-1500000) | 0.89 |
| SLC17A3 | 5.23 | 10.397 | 7.796 | -1.267 | 0.002 | 7.1 (1.4-36) | 0.02 |
| SV2B | 135.947 | 183.297 | 159.465 | -0.431 | 0.299 | 1.4 (0.9-2.1) | 0.14 |
| SLC2A6 | 716.308 | 270.812 | 495.035 | 1.403 | <0.001 | 1.2 (0.96-1.5) | 0.11 |
| SLC2A10 | 66.542 | 93.563 | 79.963 | -0.491 | 0.221 | 1.2 (0.82-1.9) | 0.31 |
| MFSD12 | 2688.538 | 2137.565 | 2414.876 | 0.331 | 0.002 | 2 (1.3-3.3) | 0.003 |
| MFSD9 | 383.42 | 405.946 | 394.608 | -0.082 | 0.252 | 1.1 (0.54-2.3) | 0.77 |
| SLC22A13 | 24.808 | 21.529 | 23.179 | 0.207 | 0.2 | 0.69 (0.21-2.3) | 0.55 |
| SLC22A14 | 3.808 | 3.936 | 3.871 | -0.037 | 0.911 | 1.7 (0.0048-570) | 0.87 |
| SLC16A12 | 2.453 | 1.563 | 2.011 | 0.651 | 0.036 | 340 (0.15-780000) | 0.14 |
| MFSD8 | 747.452 | 855.172 | 800.955 | -0.194 | 0.005 | 0.41 (0.23-0.72) | 0.002 |
| SLC2A8 | 157.06 | 131.818 | 144.523 | 0.252 | 0.385 | 1.6 (1.2-2.3) | 0.004 |
| SLC16A1 | 721.344 | 1129.588 | 924.114 | -0.647 | 0.001 | 1.1 (0.86-1.4) | 0.47 |
| SLC22A10 | 0.98 | 2.665 | 1.817 | -1.027 | 0.069 | 0.83 (0.038-18) | 0.9 |
| SLC22A17 | 57.531 | 99.47 | 78.362 | 0.042 | 0.919 | 1 (0.7-1.4) | 0.99 |
| MFSD11 | 1088.522 | 966.445 | 1027.888 | 0.172 | 0.001 | 0.87 (0.4-1.9) | 0.72 |
| SLC46A1 | 204.748 | 173.908 | 189.43 | 0.235 | 0.198 | 1.7 (0.92-3.1) | 0.089 |
| UNC93B1 | 3206.344 | 1286.753 | 2252.905 | 1.317 | <0.001 | 1.6 (1.2-2) | <0.001 |
| SLC2A9 | 714.257 | 321.801 | 519.328 | 1.15 | <0.001 | 1.2 (0.92-1.6) | 0.18 |
| SLC16A14 | 45.585 | 70.672 | 58.045 | -0.376 | 0.154 | 0.77 (0.46-1.3) | 0.31 |
| SLC16A3 | 3152.446 | 1558.668 | 2360.834 | 1.016 | <0.001 | 1.3 (1-1.7) | 0.021 |
| MFSD7 | 333.565 | 146.539 | 240.671 | 1.187 | <0.001 | 1.3 (1-1.7) | 0.052 |
| SLC16A11 | 24.049 | 18.586 | 21.335 | 0.375 | 0.052 | 1.5 (0.66-3.6) | 0.31 |
| SLC22A1 | 11.751 | 3.421 | 7.614 | 1.778 | <0.001 | 2.9 (1-8.5) | 0.05 |
| SV2A | 1021.806 | 1625.991 | 1321.898 | -0.67 | 0.004 | 1.1 (0.94-1.4) | 0.17 |
| SLC2A5 | 2235.116 | 2095.938 | 2165.988 | 0.093 | 0.784 | 1.4 (1.2-1.7) | <0.001 |
| SLC22A16 | 940.624 | 1309.556 | 1123.868 | -0.477 | <0.001 | 0.66 (0.5-0.88) | 0.005 |
| FLVCR1 | 968.057 | 1163.067 | 1064.916 | -0.265 | <0.001 | 0.84 (0.46-1.5) | 0.57 |
| FLVCR2 | 480.313 | 183.017 | 332.65 | 1.391 | <0.001 | 1.4 (1-1.8) | 0.041 |
| SLC16A8 | 134.512 | 112.188 | 123.424 | 0.261 | 0.34 | 1.7 (1.3-2.2) | <0.001 |
| SLC22A4 | 500.331 | 453.559 | 477.1 | 0.142 | 0.485 | 0.74 (0.56-0.97) | 0.027 |
| SLC22A7 | 16.171 | 12.562 | 14.379 | 0.365 | 0.244 | 1.6 (0.69-3.6) | 0.29 |
| SLC22A8 | 1.176 | 2.031 | 1.601 | -0.813 | 0.006 | 0.031 (4.8e-11-2e+07) | 0.74 |
| SLC17A5 | 672.377 | 530.559 | 601.938 | 0.341 | <0.001 | 1.2 (0.68-2.1) | 0.53 |
| SLC17A8 | 0.634 | 0.556 | 0.595 | 0.154 | 0.801 | 1.2 (9.3e-10-1.6e+09) | 0.99 |
| SLC2A12 | 34.272 | 31.422 | 32.856 | 0.73 | 0.06 | 0.58 (0.29-1.1) | 0.11 |
| SVOP | 2.35 | 2.212 | 2.281 | 0.077 | 0.809 | 0.00018 (2.4e-12-14000) | 0.35 |
| SLC17A2 | 3.719 | 77.49 | 40.36 | -4.251 | <0.001 | 1.3 (0.94-1.7) | 0.12 |
